# Role of Daptomycin in the Induction and Persistence of the Viable but Non-Culturable State of *Staphylococcus Aureus* Biofilms

**DOI:** 10.3390/pathogens3030759

**Published:** 2014-09-18

**Authors:** Sonia Pasquaroli, Barbara Citterio, Andrea Di Cesare, Mehdi Amiri, Anita Manti, Claudia Vuotto, Francesca Biavasco

**Affiliations:** 1 Department of Life and Environmental Sciences, Polytechnic University of Marche, Ancona 60131, Italy; E-Mails: s.pasquaroli@univpm.it (S.P.); andrix.di.cesare@alice.it (A.D.C.); amiri.m1983@gmail.com (M.A.); 2 Department of Biomolecular Sciences, Sect. Toxicological, Hygiene, and Environmental Sciences, University of Urbino Carlo Bo, Urbino 61029, Italy; E-Mail: barbara.citterio@uniurb.it; 3 Department of Earth, Life and Environmental Sciences, University of Urbino Carlo Bo, Urbino 61029, Italy; E-Mail: anita.manti@uniurb.it; 4 Microbial Biofilm Laboratory, IRCCS Fondazione Santa Lucia, Rome 00179, Italy; E-Mail: c.vuotto@hsantalucia.it

**Keywords:** *Staphylococcus aureus* biofilm, VBNC, daptomycin

## Abstract

We have recently demonstrated that antibiotic pressure can induce the viable but non-culturable (VBNC) state in *Staphylococcus aureus* biofilms. Since dormant bacterial cells can undermine anti-infective therapy, a greater understanding of the role of antibiotics of last resort, including daptomycin, is crucial. Methicillin-resistant *S. aureus* 10850 biofilms were maintained on non-nutrient (NN) agar in the presence or absence of the MIC of daptomycin until loss of culturability. Viable cells were monitored by epifluorescence microscopy and flow cytometry for 150 days. All biofilms reached non-culturability at 40 days and showed a similar amount of viable cells; however, in biofilms exposed to daptomycin, their number remained unchanged throughout the experiment, whereas in those maintained on NN agar alone, no viable cells were detected after 150 days. Gene expression assays showed that after achievement of non-culturability, 16S rDNA and *mecA* were expressed by all biofilms, whereas *glt* expression was found only in daptomycin-exposed biofilms. Our findings suggest that low daptomycin concentrations, such as those that are likely to obtain within biofilms, can influence the viability and gene expression of non-culturable *S. aureus* cells. Resuscitation experiments are needed to establish the VBNC state of daptomycin-exposed biofilms.

## 1. Introduction

Biofilm production protects bacteria from a number of stress conditions [1,2], it promotes antibiotic resistance [2,3] and is often related to the onset of persistent infections [1,2], especially those associated with indwelling medical devices [3,4].

The continuous increase in antimicrobial resistance hampers the treatment of infections. The success of multidrug-resistant Gram-positive pathogens, such as methicillin-resistant *Staphylococcus aureus* (MRSA) [5,6,7], vancomycin-resistant enterococci (VRE) [8] and coagulase-negative staphylococci [9], emphasizes the need for new antimicrobials with alternative mechanisms of action. Daptomycin is a cyclic anionic lipopeptide antibiotic produced by *Streptomyces roseosporus* that, in the EU, has been approved to treat skin and soft-tissue infections since 2006 [10]. Daptomycin has bactericidal activity against Gram-positive bacteria, including MRSA [11,12] and VRE [13], and is currently the last line of defense against severe Gram-positive infections. It has a unique, but not completely elucidated, mechanism of action, where a calcium-dependent dissipation of membrane potential leads to the release of intracellular ions from the cell and, ultimately, to death [14]. Daptomycin is effective in treating skin infections, endocarditis and bacteremia [15] and in counteracting biofilm-based infections associated with medical devices [16,17], which frequently require combination therapy [18]. The combination with rifampicin or beta-lactams has been reported to be effective in treating biofilm-related enterococcal [18] and staphylococcal [12] infections.

The viable but non-culturable (VBNC) state is a survival strategy characterized by low-level metabolic activity and bacterial growth failure on standard media [19]. It protects bacterial cells from environmental stress, such as nutrient depletion, changes in temperature, pH or salinity [20], and presence of antibiotics [21]. The VBNC state has been reported for several human pathogens [19], including biofilm-producing staphylococci [21]. The critical feature of cells in the VBNC state is their ability to regain culturability in the presence of resuscitation-promoting factors [22]. In a recent *in vitro* study by our group, vancomycin and quinupristin-dalfopristin, which are often used to treat biofilm-associated chronic infections [23], have been demonstrated to promote the emergence of persistent VBNC forms in *S. aureus* biofilms [22]. These findings prompted us to establish whether daptomycin, which is considered as a last line of defense antibiotic, also induces the VBNC state. 

## 2. Results and Discussion

### 2.1. Biofilm Production, Stress Exposure and Non-Culturability

The strong biofilm producer, *S. aureus* 10850 [21,24], was analyzed for susceptibility to daptomycin by MIC determination and showed low-level resistance with a MIC value of 2 µg/mL (European Committee on Antimicrobial Susceptibility Testing (EUCAST) susceptibility breakpoint ≤1 µg/mL).

To induce the VBNC state, *S. aureus* 10850 biofilms developed on membrane filters were placed on non-nutrient (NN) agar plates without or with daptomycin (at a concentration equal to the MIC) to induce the largest possible amount of VBNC cells [21] and incubated at 37 °C. Culturability was tested every two days. 

All biofilms reached non culturability in 40 days. They were then detached from the filters, stained by the live/dead method, and examined for viable cells by epifluorescence microscopy. All filters were culture-negative and contained green coccoid cells whose average counts were 3.8 × 10^4^/mL (starved biofilms) and 2.7 × 10^4^/mL (starved + daptomycin-exposed biofilms). 

### 2.2. Persistence of the VBNC State

**Figure 1:**
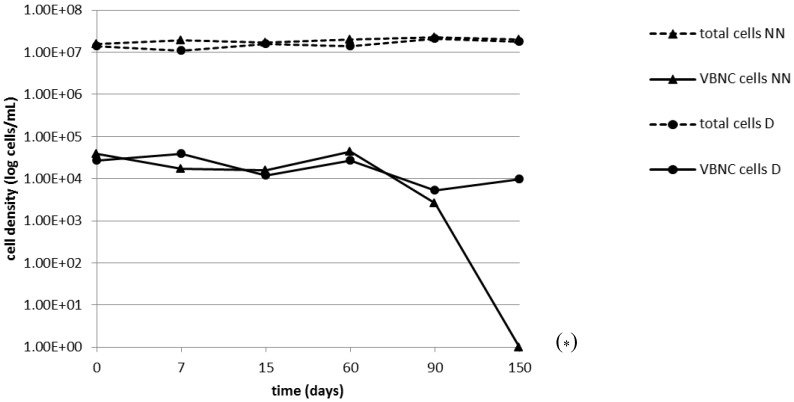
Epifluorescence counts after live-dead staining of viable and total cells in detached non-culturable *S. aureus* 10850 biofilms exposed to nutrient depletion with (D) or without (NN) daptomycin. Counts were performed at various intervals (0–150 days) from the loss of culturability. The results are the means of two counts. VBNC, viable but non-culturable.

After achievement of non-culturability on the 40th day, the persistence of the VBNC state was monitored by epifluorescence microscopy for 150 days in biofilms maintained on NN agar plates without or with daptomycin (Figure 1). Live/dead staining of a non-culturable daptomycin-exposed biofilm on the 15th day followed by flow cytometry demonstrated a viable subpopulation (Table 1), whose abundance was comparable to that documented by epifluorescence counts (differences ≤0.5 log).

The number of viable cells remained substantially unchanged throughout the experiment in starved and daptomycin-exposed biofilms; in those maintained on NN agar alone, it did not change significantly over the first 90 days, but no viable cells were left on the 150th day (Figure 1, Table 1). 

These findings indicate that daptomycin exposure extended the viability of non-culturable cells compared with starvation alone, whereas nutrient depletion rather seemed to give rise to premortem VBNC forms, supporting previous data by our group [21].

* No viable cells detected by epifluorescence microscopy (< 60 cells/mL).

**Table 1:** Total and viable cells counts in biofilms exposed to nutrient depletion or nutrient depletion with daptomycin.

Stress Condition	Days Since Achievement of Non-Culturability	Cells/mL	
		Total	Viable
Nutrient depletion	0	1.6×10^7^	3.8 × 10^4^
	7	1.9 × 10^7^	1.7 × 10^4^
	15	1.7 × 10^7^/1.5 × 10^8 ^*	1.6 × 10^4^/8.2 × 10^4 ^*
	60	2.0 × 10^7^	4.4 × 10^4^
	90	2.3 × 10^7^	2.6 × 10^3^
	150	2.0 × 10^7^	<60
Nutrient depletion+ daptomycin	0	1.4 × 10^7^	2.7 × 10^4^
	7	1.1 × 10^7^	3.9 × 10^4^
	15	1.6 × 10^7^/1.4 × 10^8 ^*	1.2 × 10^4^/7.6 × 10^4 ^*
	60	1.4 × 10^7^	2.7 × 10^4^
	90	2.1 × 10^7^	5.3 × 10^3^
	150	1.8 × 10^7^	9.8 × 10^3^

* Flow cytometric analysis.

### 2.3. Gene Expression of VBNC Cells

The presence of viable subpopulations after loss of culturability was tested by gene expression experiments. Non-culturable biofilms, either starved and starved and daptomycin-exposed, were tested for the expression of two *S. aureus* housekeeping genes, 16S rDNA and *glt* (coding for glutamate synthase). Aliquots of non-culturable biofilms detached 0, 7, 15 and 60 days from the loss of culturability were analyzed by real-time RT–PCR. The expression of 16S rRNA was detected in all biofilms tested, whereas *glt* was expressed exclusively in daptomycin-exposed biofilms at 0, 7 and 15 days (Table 2). Since 16S rRNA quantification is considered a reliable assay of viability [25], these findings support the epifluorescence and cytofluorimetric evidence of the presence of VBNC cells, in line with data from an earlier study by our group [21]. Given that glutamate synthase is involved in the incorporation of ammonium ions into organic compounds, which is a key step in amino acid production [26], it could have a role in the persistence of a true viable state in non-culturable biofilms via continued amino acid uptake and incorporation [27].

The ability of non-culturable biofilms to express virulence and antibiotic resistance genes was explored by further real-time RT-PCR assays targeting the thermonuclease (*nuc*) and the methicillin resistance penicillin binding protein 2a (PBP2a) (*mecA*) genes. *nuc* expression was detected in none of the biofilms analyzed and *mec*A expression in all of them (Table 2). These findings suggest that thermonuclease may not be essential for biofilm survival *in vitro*. Moreover, the paucity of metabolically active cells suggests that its expression could be inhibited by quorum sensing [28]. Conversely, the *mecA* gene encodes a PBP involved in cell wall formation [5] that could play an important role in maintaining the wall of VBNC cells intact and functional.

**Table 2:** Expression of key genes in non-culturable *S. aureus* 10850 biofilms exposed to nutrient depletion with or without daptomycin.

Stress Condition	Time from non Culturability	Gene Analysis			
		*16SrDNA*	*glt*	*nuc*	*mecA*
Nutrient depletion	T0	+	ND	ND	ND
	T7	+	-	-	+
	T15	+	-	-	+
	T60	+	-	-	+
Nutrient depletion + daptomycin	T0	+	ND	ND	ND
	T7	+	+	-	+
	T15	+	+	-	+
	T60	+	-	-	+

ND: not determined.

Taken together, the findings of our experiments seem to indicate that daptomycin acts as an inducer of the VBNC state of *S. aureus* by modulating gene expression, activating or repressing the transcription of specific genes. This effect may be explained by a poor penetration of daptomycin and the achievement of subinhibitory concentrations in the deep layers of the *S. aureus* biofilm matrix.

Indeed, low concentrations of a number of antibiotics exert biological activities other than inhibition, with major effects on transcription patterns [29]. The phenomenon, known as hormesis, involves biological responses to environmental signals or stress conditions that are characterized by biphasic dose-response relationships exhibiting low-dose stimulation and high-dose inhibition [30]. Subinhibitory concentrations of antimicrobial peptides, such as the cyclic lipopeptide daptomycin, which causes rapid membrane depolarization and potassium ion efflux, can thus increase the transcription levels of osmoprotectants, countering the osmotic stress, and downregulate the ribose transport system [30]. Given that ribose is a key element for ATP and RNA synthesis, this condition could be a stress factor for bacterial cells, contributing to VBNC state induction and persistence.

Since increased cell wall thickness is typical of the VBNC state [19], it may be hypothesized that low daptomycin concentrations stimulate the synthesis of peptidoglycan genes, as reported for imipenem in studies of the *Pseudomonas aeruginosa* transcriptome [30]. On the other hand, an involvement of daptomycin in the regulation of cell wall synthesis is quite likely, given that PBPs are membrane proteins. A role for daptomycin may also be inferred based on its reported synergism with beta-lactams against staphylococcal biofilms [12].

## 3. Experimental Section

### 3.1. Bacterial Strains, Media, Antibiotics and Enzymes

The strong biofilm producer, *S. aureus* 10850 [24], was routinely grown in tryptic soy broth (TSB) or agar (TSA) (Oxoid, Basingstoke, U.K.), supplemented with 1% (v/v) glucose (TSBG or TSAG) to promote biofilm production. M9 minimal medium without glucose was used as NN agar in VBNC induction assays, as described by Pasquaroli *et al*. [21]. The following antibiotics and enzymes were used: daptomycin (Cubicin, Novartis Pharma SpA, Italy) and lysozyme and lysostaphin (Sigma-Aldrich St Louis, MO, USA).

### 3.2. MIC Determination

The MIC of daptomycin was determined by a broth microdilution method, and *S. aureus* susceptibility was defined according to the European Committee on Antimicrobial Susceptibility Testing (EUCAST) breakpoints [31]. The test medium was MHII broth (Becton-Dickinson, Milan, Italy) supplemented with CaCl_2 _(calcium chloride; Merck KGaA, Darmstadt, Germany) to a final Ca^2+^ concentration of 50 µg/mL. *S. aureus* ATCC 29213 was used as the control strain.

### 3.3. Biofilm Production, Stress Exposure and Culturability Assays

*In vitro* biofilm production, stress exposure and culturability assays were performed as described by Pasquaroli *et al.* [21]. Briefly, 100 µL of a late-log culture of *S. aureus* 10850 grown in TSBG was spotted on 0.22-µm sterile nitrocellulose filters (Millipore Corporation, Billerica, MA, USA); the filters were placed onto TSAG plates for 48 h at 37 °C to allow biofilm development at the filter-air interface. They were then moved to NN agar plates, unsupplemented or supplemented with daptomycin (2 µg/mL), and incubated at 37 °C until loss of culturability. Filter cultures were transferred weekly to fresh agar plates without washing. Culturability was assessed every 2 days by placing a loop of filter cultures in TSB and onto TSA, followed by incubation for 48 h at 37 °C. Filter cultures testing negative on culturability assays were placed in 5 mL saline, detached by 3 cycles of sonication and vortexing, washed and resuspended in the same volume of saline.

### 3.4. Epifluorescence Microscopy and Flow Cytometry

Epifluorescence microscopy and flow cytometry counts were performed as described previously [21]; each count was carried out in duplicate. The limit of detection of epifluorescence counts was 60 cells/mL of detached biofilm.

### 3.5. Real-Time RT–PCR Assays

Total RNA was extracted from 5 mL of detached biofilm using the RNeasy Mini Kit (Qiagen, Hilden, Germany), as described previously [21]. Total RNA was retro-transcribed using Qiagen’s QuantiTect Reserve Transcription kit.

Real-time PCRs were carried out in a total volume of 20 µL containing 0.25 µM of each primer (Table 3), 10 µL of 2× Supermix (Qiagen) and 2 µL of reverse transcription mixture. Cycling conditions were 95 °C for 3 min, followed by 40 cycles of 95 °C for 10 s, different annealing temperatures (Table 3) for 20 s and 72 °C for 20 s. Amplification reactions and melt-curve analysis were performed using the Rotor-Gene Q MDx (Qiagen). cDNA obtained starting from a broth culture of *S. aureus* 10850 was used as a positive control.

**Table 3:** Target genes and primer pairs used in gene expression assays.

Target Gene	Gene Function	Primer Pair (5ʹ-3ʹ)	Annealing Temperature (°C)	Product Size (bp)	Reference
*16S rDNA*	Housekeeping	F-TGGAGCATGTGGTTTAATTCGA R-TGCGGGACTTAACCCAACA	60	159	[32]
*glt*	Species specific, coding for glutamate synthase	F-AATCTTTGTCGGTACACGATATTCTTCACG R-CGTAATGAGATTTCAGTAGATAATACAACA	58	108	[33]
*nuc*	Virulence factor, coding for thermonuclease	F-GACTATTATTGGTTGATCCACCTG R- GCCTTGACGAACTAAAGCTTCG	60	218	[34]
*mecA*	Methicillin resistance	F-TCCAGATTACAACTTCACCAGG R-CCACTTCATATCTTGTAACG	57	162	[35]

## 4. Conclusions

The present findings provide evidence that daptomycin may play a role in the induction and persistence of VBNC *S. aureus* biofilms by showing cell viability and gene expression for months after achievement of non-culturability. Infections caused by staphylococcal biofilms should be treated by carefully selected and dosed antibiotics, to maintain drug concentrations capable of exerting full inhibitory activity. The ability of non-culturable daptomycin-exposed staphylococcal biofilms to resuscitate requires additional testing. Further experiments are under way in our laboratory.
